# Chronic Kidney Disease in a Large National Human Immunodeficiency Virus Treatment Program

**DOI:** 10.3390/healthcare10081490

**Published:** 2022-08-08

**Authors:** Ninutcha Paengsai, Kajohnsak Noppakun, Gonzague Jourdain, Tim Roy Cressey, Nicolas Salvadori, Romanee Chaiwarith, Apichat Tantraworasin, Jean Yves Mary, Chureeratana Bowonwatanuwong, Sorakij Bhakeecheep, Patrinee Traisathit, Natapong Kosachunhanun

**Affiliations:** 1 Clinical Epidemiology Program, Faculty of Medicine, Chiang Mai University, Chiang Mai 50200, Thailand; 2 National Health Security Office (NHSO), Bangkok 10210, Thailand; 3 Clinical Epidemiology and Clinical Statistics Center, Faculty of Medicine, Chiang Mai University, Chiang Mai 50200, Thailand; 4 Division of Nephrology, Department of Internal Medicine, Faculty of Medicine, Chiang Mai University, Chiang Mai 50200, Thailand; 5 Pharmacoepidemiology and Statistics Research Center (PESRC), Faculty of Pharmacy, Chiang Mai University, Chiang Mai 50200, Thailand; 6 Institut de Recherche pour le Développement (IRD), MIVEGEC, 13002 Marseille, France; 7 Faculty of Associated Medical Sciences, Chiang Mai University, Chiang Mai 50200, Thailand; 8 Data Science Research Center, Department of Statistics, Faculty of Science, Chiang Mai University, Chiang Mai 50200, Thailand; 9 Department of Molecular and Clinical Pharmacology, University of Liverpool, Liverpool L69 3GF, UK; 10 Department of Statistics, Faculty of Science, Chiang Mai University, Chiang Mai 50200, Thailand; 11 Division of Infectious Disease and Tropical Medicine, Department of Internal Medicine, Faculty of Medicine, Chiang Mai University, Chiang Mai 50200, Thailand; 12 Clinical Surgical Research Center, Chiang Mai University, Chiang Mai 50200, Thailand; 13 INSERM UMR 1135, Equipe ECSTRRA, Centre de Recherche Epidémiologie Statistique Sorbonne Paris Cité, Université Paris Diderot, 75004 Paris, France; 14 Department of Internal Medicine, Chonburi Hospital, Chonburi 20000, Thailand; 15 School of Medicine, University of Phayao, Phayao 56000, Thailand; 16 Division of Endocrinology, Department of Internal Medicine, Faculty of Medicine, Chiang Mai University, Chiang Mai 50200, Thailand

**Keywords:** antiretroviral therapy, chronic kidney disease, HIV infection, tenofovir disoproxil fumarate

## Abstract

Tenofovir disoproxil fumarate (TDF) is associated with a risk of chronic kidney disease (CKD), especially in Asian populations. Data from the Thai national health insurance system was used to assess CKD incidence in patients receiving antiretroviral therapy in real-world practice. We analyzed data from patients who initiated one of the following first-line regimens: zidovudine + lamivudine + nevirapine (AZT + 3TC + NVP); zidovudine + lamivudine + efavirenz (AZT + 3TC + EFV); tenofovir + lamivudine + nevirapine (TDF + 3TC + NVP); tenofovir + lamivudine/emtricitabine + efavirenz (TDF + 3TC/FTC + EFV); and tenofovir +lamivudine +lopinavir/ritonavir (TDF + 3TC + LPV/r). CKD was defined as glomerular filtration rate <60 mL/min/1.73 m^2^ for >3 months, or a confirmed 2010 WHO diagnosis (ICD-10 code N183, N184, or N185). Death competing risk survival regression models were used. Among 27,313 participants, with a median age of 36.8 years and median follow-up of 2.3 years, 245 patients (0.9%) were diagnosed with CKD (incidence 3.2 per 1000 patient-years; 95% CI 2.8–3.6). Compared with patients receiving AZT + 3TC + NVP, the risk of CKD measured by adjusted sub-distribution hazard ratio (aSHR) was 6.5 (95% CI 3.9–11.1) in patients on TDF + 3TC + LPV/r, 3.8 (95% CI 2.3–6.0) in TDF + 3TC + NVP, and 1.6 (95% CI 1.2–2.3) in TDF + 3TC/FTC + EFV. Among patients receiving TDF, compared with those receiving TDF + 3TC/FTC + EFV, the aSHR was 4.0 (95% CI 2.3–6.8) in TDF + 3TC + LPV/r and 2.3 (95% CI 1.4–3.6) in TDF + 3TC + NVP. TDF was associated with an increased risk of CKD, especially when combined with LPV/r or NVP.

## 1. Introduction

The Global Burden of Disease estimated that 1.2 million people died from chronic kidney disease (CKD) in 2017 [1]. With an improvement in the access to antiretroviral therapy (ART), the causes of death in human immunodeficiency virus (HIV)-infected individuals are shifting from infectious diseases to non-communicable diseases, including several conditions associated with the use of some antiretroviral agents. CKD has emerged as an important problem in HIV-infected individuals [2,3,4]. A large meta-analysis of studies in HIV-infected adults from over 60 countries showed that the overall prevalence of CKD defined by an estimated glomerular filtration rate (eGFR) <60 mL/min/1.73 m^2^ was 4.8% and 19.4% of patients with diabetes mellitus [5]. High levels of viral loads, low CD4 cell counts, diagnosis of acquired immunodeficiency syndrome (AIDS), low body weight, and the use of some antiretroviral agents, such as indinavir, tenofovir disoproxil fumarate (TDF), and ritonavir-boosted lopinavir (LPV/r), are well-documented risk factors for CKD in HIV-infected individuals [6,7,8,9]. Several studies have demonstrated an increased risk of CKD in patients receiving TDF, especially in combination with protease inhibitors, such as lopinavir/ritonavir. However, little is known about the actual burden of this problem in relation to first-line antiretroviral combinations used in adults within large treatment programs in the real world, especially in Asia where the bodyweight of adults with HIV tends to be lower than those in other high settings.

Since 2004, the Thailand National Health Security Office (NHSO) in charge of the National AIDS Program has provided free-of-charge health services and ART for HIV-infected individuals. Using data from adults registered with this program under the Universal Coverage Scheme (UCS) [10], we investigated the association between the incidence of CKD in adults with HIV and first-line ART regimens to help to inform clinicians when selecting a first-line regimen recommended by the 2019 World Health Organization guidelines [11].

## 2. Materials and Methods

### 2.1. Study Design and Participants

We performed a retrospective cohort study using data from the National AIDS Program, covering HIV-infected individuals who received ART. This program is under the Universal Coverage Scheme (UCS), which covers about three-quarters of the Thai population (~47 millions). Participants eligible for inclusion were as follows: (i) aged 18 years or older having registered for ART between October 2006 and September 2013 (Fiscal Years (FY) 2007 to 2013); (ii) received a first-line ART regimen recommended by the 2019 World Health Organization guideline [11]; and (iii) did not have, when they initiated treatment, chronic kidney disease (CKD) defined by the estimated glomerular filtration rate (eGFR) (estimated using the Chronic Kidney Disease Epidemiology Collaboration equation [12]) and ICD-10 code. We excluded participants who did not receive ART or had incomplete baseline or follow-up information.

### 2.2. Exposure

The exposure of interest was one of the first-line ART regimens: (i) zidovudine, lamivudine, and nevirapine (AZT + 3TC + NVP); (ii) zidovudine, lamivudine, and efavirenz (AZT + 3TC + EFV); (iii) tenofovir, lamivudine, and nevirapine (TDF + 3TC + NVP); (iv) tenofovir, lamivudine or emtricitabine, and efavirenz (TDF + 3TC/FTC + EFV); and (v) tenofovir, lamivudine, and lopinavir/ritonavir (TDF + 3TC + LPV/r) [10].

### 2.3. Outcomes

The outcome of interest was the diagnosis (confirmed within 3 months) of conditions defined by the 2010 WHO ICD-10 [13] as codes N183, N184, N185, i.e., CKD stage 3, 4, or 5 (kidney damage with moderately to severely decreased GFR [15–59 mL/min] or chronic uremia or end-stage kidney disease [13]); or eGFR < 60 mL/min/1.73 m^2^ for >3 months [14].

We used patients’ data from the date of ART initiation to last visit, switching of ART regimen, loss to follow up, end of study (30 September 2014), death, or confirmed diagnosis of CKD, whichever occurred first.

### 2.4. Variables

We extracted the following variables at time of ART initiation: sex, age, weight, height, serum creatinine levels, fasting plasma glucose, serum triglycerides, serum total cholesterol, absolute CD4 cell count, HIV-1 ribonucleic acid (RNA) load, and history of comorbidities, including type 2 diabetes, hypertension, ischemic heart disease, tubulo-interstitial nephritis, gout, urolithiasis, hepatitis B virus (HBV) infection, and hepatitis C virus (HCV) infection.

We considered that a patient was diagnosed with type 2 diabetes if either of the following records were available: confirmed fasting plasma glucose ≥126 mg/dL according to the 2013 American Diabetes Association criteria [15], or WHO diagnosis (ICD-10 code E11–E14) [13], or receipt of anti-diabetic drugs at least twice of medical services. Hyperlipidemia was defined by either total cholesterol ≥240 mg/dL or triglycerides ≥200 mg/dL [16]. Hypertension was defined by a confirmed WHO diagnosis (ICD-10 code I10–I15) or receipt of anti-hypertensive drugs. WHO ICD-10 was used to identify history of comorbidities: ischemic heart disease (codes I20–I25), tubulo-interstitial nephritis (codes N10–N12), gout (code M10), urolithiasis (codes N20–N23), HBV infection (codes B16, B170, B180 and B181), and HCV infection (codes B171 and B182) [17].

### 2.5. Statistical Analyses

The characteristics of participants in the study are presented as medians and interquartile ranges (IQR) for continuous variables, and as counts and percentages for categorical variables. The number of person-years of follow-up (PYFU) was calculated from the date of ART initiation to censoring date. For descriptive purposes, the overall CKD incidence rate was estimated by the number of newly diagnosed individuals divided by the total number of PYFU, and the 95% confidence interval (CI) was calculated using the quadratic approximation to the Poisson log-likelihood.

Regarding missing data, all models were adjusted for the availability of serum creatinine measurement records at baseline and during follow-up. We imputed missing data by multiple imputation with chained equations (MICE) based on logistic regression for variables with ≤20% missing data, and for time-updated absolute CD4 cell count values if data were missing for more than 9 months after the previous known value [18]. Baseline body mass index and time-updated HIV-1 RNA load were excluded from the main analysis because more than 20% of the values were missing.

A propensity score for each first-line ART regimen was developed to minimize the possible bias of the choice of ART by the physician in charge, and all models were stratified by propensity score. This score was generated using multinomial logistic regression based on a fiscal year of ART initiation, sex, age group, and history of comorbidities (HBV and HCV infection, hypertension, type 2 diabetes, ischemic heart disease, tubulo-interstitial nephritis, gout, and urolithiasis) (see Supplementary Materials Figure S1).

We estimated the cumulative incidence rate of CKD and corresponding 95% CI using a cumulative incidence function accounting for deaths without a prior CKD diagnosis as competing events [19]. 

The following variables potentially associated with risk of CKD were considered for analysis: fiscal year of ART initiation, sex, age categories (18–34, 35–44, 45–59, or ≥60 years), body mass index (<18.5, 18.5–22.9, 23.0–24.9, or ≥25 kg/m^2^), and history of comorbidities at treatment initiation, time updated absolute CD4 cell count (<200 or ≥200 cells/mm^3^), and HIV-1 RNA load (<1000 or ≥1000 copies/mL). 

We analyzed the association between first-line ART regimens and risk of CKD using Fine and Gray competing risks survival regression [20,21]. Variables potentially associated with the risk of CKD (listed above) were tested in univariable models adjusting for potential confounders. We retained those associated at the p-value of 0.20 or less for multivariable analyses. We used a backward elimination approach regression analysis using the Wald chi-squared test starting to define the final model. The associations were expressed in adjusted sub-distribution hazard ratio (aSHR). 

All analyses were performed using Stata software, version 15.1 (Stata Corp, College Station, TX, USA).

## 3. Results

### 3.1. Study Population and Follow-Up

Of 152,664 HIV-infected adults, 100,081 were excluded from the study because their first-line ART regimens were not recommended by WHO guidelines, in addition to 25,270 patients because of insufficient data. Thus, a total of 27,313 patients were included in this analysis, and 15,389 (56.3%) were males.

**Figure 1:**
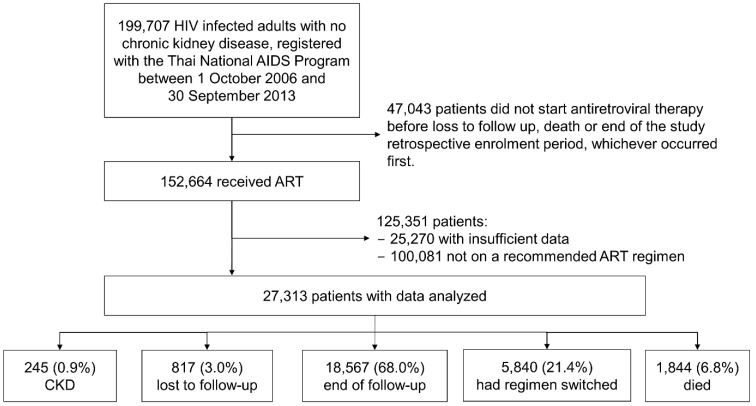
Patients’ disposition: reasons for patient selection for retrospective participation in the study analyses. Abbreviations: AIDS, acquired immunodeficiency syndrome; ART, antiretroviral therapy; CKD, chronic kidney disease; HIV, human immunodeficiency virus.

Over the 8-year study period, of these 27,313 patients, 817 (3.0%) were lost to follow-up, 5840 (21.4%) switched to a second-line ART regimen, and 1844 (6.8%) died (Figure 1). The median duration of follow-up for the first-line ARV drug regiment was 2.3 years (IQR 1.5–3.6), accounting for a total 76,168 PYFU.

At baseline, the median age was 36.8 years (IQR, 30.8–43.3), body mass index 20.4 kg/m^2^ (IQR, 18.6–22.6), and absolute CD4 cell count 146 cells/mm^3^ (IQR, 49–244). The most common comorbidities were hypertension (3.9%), HBV infection (3.8%), tubulo-interstitial nephritis (1.9%), and DM (1.9%). Table 1 shows the distribution of baseline characteristics overall and by ART regimen.

### 3.2. Incidence of CKD

During the total 76,168 PYFU, of 27,313 adults, 245 (0.9%) were diagnosed with CKD, leading to a cumulative incidence of 3.2 per 1000 PYFU (95% CI 2.8–3.6). The incidence rate of CKD was lower during the first 2 years of follow-up compared to the incidence thereafter (see Supplementary Materials Table S1).

The incidence rate of CKD was higher in adults who received a TDF containing ART regimen (5.2 per 1000 PYFU; 95% CI 4.4–6.3) compared to those receiving a non-TDF containing ART regimen (2.3 per 1000 PYFU; 95% CI 2.0–2.8) (Table 2).

The estimated cumulative incidence of CKD at 8 years of follow-up was 1.9% (95% CI 1.4–2.3). It was 1.5% (95% CI 0.1–2.2) in patients on AZT + 3TC + NVP, 2.8% (95% CI 1.5–4.9) on TDF + 3TC/FTC + EFV, 4.0% (95% CI 2.6–5.9) on TDF + 3TC + NVP, 1.3% (95% CI 0.9–2.0) on AZT + 3TC + EFV, and 5.1% (95% CI 3.0–8.1) on TDF + 3TC + LPV/r.

### 3.3. Association between First-Line Antiretroviral Treatment Regimen and Chronic Kidney Disease

In univariable analyses, the risk of CKD diagnosis was associated with older age at baseline, history of type 2 diabetes, hypertension, tubule-interstitial nephritis, gout and urolithiasis at baseline, and time-updated absolute CD4 cell count <200 cells/mm^3^ (all *p* ≤ 0.05, Table 3).

**Figure 2:**
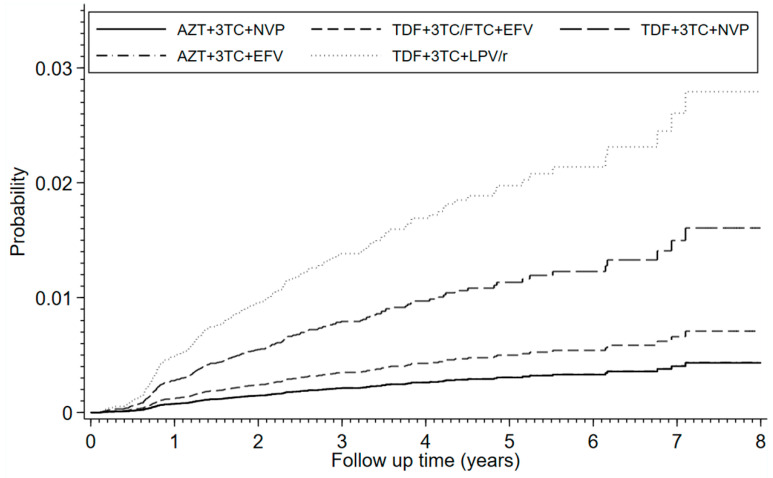
Cumulative incidence function of chronic kidney disease, accounting for deaths without prior CKD diagnosis as competing events. Analyses were adjusted for sex, age, history of type 2 diabetes, hypertension, gout, urolithiasis, serum creatinine levels, and propensity score stratification. The follow-up time was from ART initiation until last visit, switching of ART regimen, loss to follow up, end of study, death or confirmed diagnosis of CKD, whichever occurred first. The overall cumulative incidence rates were: 0.0036 (95% CI 0.0030–0.0044) at 1 year of follow-up, 0.0071 (0.0061–0.0083) at 2 years, 0.0101 (0.0088–0.0116) at 3 years, 0.0120 (0.0104–0.0138) at 4 years, 0.0137 (0.0117–0.0158) at 5 years, 0.0146 (0.0124–0.0170) at 6 years, 0.0174 (0.0140–0.0213) at 7 years, and 0.0185 (0.0146–0.0230) at 8 years.

In the final model adjusting for sex, age, history of type 2 diabetes, hypertension gout, and urolithiasis at baseline, time-update absolute CD4 cell count <200 cells/mm^3^, availability of previous serum creatinine measurement and propensity score stratification, and compared to AZT + 3TC + NVP, the following three regimens were associated with a higher risk of CKD: TDF + 3TC + LPV/r (aSHR 6.5, 95% CI 3.9–11.1), TDF + 3TC + NVP (aSHR 3.8, 95% CI 2.3–6.0), and TDF + 3TC/FTC + EFV (aSHR 1.6, 95% CI 1.2–2.3) (Table 3). Among patients receiving TDF, compared to TDF + 3TC/FTC + EFV, the risk was higher on TDF + 3TC + LPV/r (aSHR 4.0, CI 2.3–6.8) or TDF + 3TC + NVP (aSHR 2.3, CI 1.4–3.6). The estimated cumulative incidence rates by ART regimen are presented in Figure 2. 

## 4. Discussion

Using data from 27,313 HIV-infected adults on ART over a median follow up of 2.3 years (76,168 PYFU), the estimated incidence rate of CKD was 3.2 per 1000 PYFU. Factors independently associated with CKD diagnosis were older age, type 2 diabetes, gout and urolithiasis, which are known risk factors in the general population [22,23,24,25], receipt of TDF-containing regimens, and absolute CD4 cell count <200 cells/mm^3^, both being factors related to HIV infection.

The Data Collection on Adverse Events of Anti-HIV Drugs (D:A:D) study in Europe, the USA, and Australia reported a CKD incidence rate of 1.8 per 1000 PYFU (95% CI 1.6–2.0) in HIV-positive adults [26], lower than in our study. However, in our study, data on atazanavir and abacavir were too scarce to be analyzed and compared to D:A:D study Two studies in Asia reported higher CKD incidence rates than in our study. The HIV Netherlands Australia Thailand Research Collaboration (HIV-NAT) reported an overall CKD incidence rate of 10.4 per 1000 PYFU, but no breakdown by ART regimen was provided, limiting the possibility to compare [27]. The AIDS Clinical Center of National Center for Global Health and Medicine (NCGM) in Japan reported an overall CKD incidence rate of 20.6 per 1000 PYFU (95% CI 17.6–24.2), probably due to a high percentage of patients (83%) on ritonavir-boosted protease inhibitors [28]. 

In our population, older age, T2DM, and hypertension were associated with the risk of CKD, as in the general population [28,29,30,31]. HBV and HCV co-infections were not associated with the risk of CKD in our study, although an association with HBV was found in other studies [32,33] and with HCV [8].

Two studies in Africa reported that the risk of CKD preferably occurred during the first 3 years following HIV diagnosis and in the case of low CD4 cell counts [34,35]. In the INSIGHT Strategic Timing of AntiRetroviral Treatment (START) trial, ART-naïve patients with CD4 cell counts ≤500 cells/μL had a higher prevalence of CKD than those with higher CD4 cell counts [29]. We also found an association with lower CD4 cell counts. An increased HIV-1 RNA load has also been reported as a significant risk factor [27], but we could not analyze this association due to insufficient baseline HIV-1 RNA load data.

We found that TDF exposure increased the risk of CKD, as in the EuroSIDA Study Group [36], the U.S. veterans study [37], a prospective cohort from France [38], the Canadian HIV Observational Cohort study [8], and the D:A:D study [26]. However, the NCGM study found no increase in the incidence of CKD among patients on TDF (despite a median observation duration of 5.1 years), but a higher drop in eGFR with longer exposure to TDF [28]. Interestingly, a retrospective study in Kenya [39] raised the question of a potential risk of CKD associated with NVP when in combination with TDF. To our knowledge, only one other study, also in Thailand, showed that patients on TDF + 3TC + NVP had a higher risk of renal impairment compared to patients on TDF + 3TC + EFV [40]. It is unclear whether this could be explained by confounding factors that were not taken into account.

As for the use of protease inhibitor, the Canadian HIV Observational Cohort study [8] and the D:A:D study [26] reported that the exposure to LPV/r was significantly associated with an increased risk of CKD, as in our study. In HIV-positive adults with kidney dysfunction, plasma TDF concentration in peripheral blood mononuclear cells has been shown to be significantly higher among patients receiving LPV/r compared with those receiving an NNRTI (NVP or EFV) [41]. A recent study from the USA National Historical Cohort of HIV-infected Veterans reported that an incidence rate of CKD in patients on EFV + FTC + TDF of 39.3 per 1000 PYFU (95% CI 34.0–45.3), significantly lower when compared to ritonavir-boosted PI (atazanavir, LPV, or darunavir) +FTC + TDF (66.1 per 1000 PYFU, 95% CI 55.7–77.9) (HR 0.6, 95% CI 0.5–0.7) [42].

In our study, attending physicians switched the regimens of 5840 (21.4%) patients due to virologic failure or side effects. The choice of the new regimen was guided by HIV-1 genotypic resistance test results, adherence, potential drug–drug interactions, relevant comorbidities, drug availability in the national program, and national guidelines. The cost of laboratory exams and drugs were not supported by the patients but by The National AIDS Program.

There were several limitations in our study. The NAP database was primarily designed to facilitate the reimbursement of costs incurred by hospitals for the delivery of HIV-related medical services, and risk factors for CKD were not systematically recorded. This study was a retrospective analysis of data collected in a real-world setting and the choice of an ART regimen was not randomized, but made by attending physicians based on baseline characteristics and guidelines. The use of a propensity score may partly correct this bias, but cannot totally eliminate it. Another limitation is that some variables, such as laboratory results at ART initiation, were not recorded in the database (cholesterol, triglycerides, and HIV-1 RNA load) and could not be fully assessed in our analysis. Nevertheless, this national database provides a unique source of information reflecting the actual CKD burden in people living with HIV.

## 5. Conclusions

Based on the analysis of a large national dataset from HIV-infected patients treated in the real world, the risk of CKD was relatively low overall and mostly concentrated in patients receiving TDF with a higher incidence in those also on NVP or LPV/r. The results of our study support the current recommendation of dose adjustment in patients with kidney function impairment.

## Figures and Tables

**Table 1:** Baseline characteristics of HIV-infected adults who received the recommended first-line antiretroviral therapy regimens.

Baseline Characteristics	Antiretroviral Therapy Regimens					
	AZT + 3TC + NVP	AZT + 3TC + EFV	TDF + 3TC + NVP	TDF + 3TC/FTC + EFV	TDF + 3TC + LPV/r	Overall
	(n = 12,084)	(n = 6519)	(n = 888)	(n = 7351)	(n = 471)	(n = 27,313)
**Fiscal year of** **antiretroviral** **initiation**						
2007	757 (6.3%)	515 (7.9%)	59 (6.6%)	165 (2.2%)	27 (5.7%)	1523 (5.6%)
2008	823 (6.8%)	477 (7.3%)	55 (6.2%)	228 (3.1%)	39 (8.3%)	1622 (6.0%)
2009	901 (7.5%)	588 (9.0%)	78 (8.8%)	358 (4.9%)	46 (9.8%)	1971 (7.2%)
2010	1193 (9.9%)	726 (11.1%)	93 (10.5%)	667 (9.1%)	54 (11.5%)	2733 (10.0%)
2011	2656 (22.0%)	1295 (19.9%)	190 (21.4%)	1484 (20.2%)	88 (18.7%)	5713 (20.9%)
2012	2639 (21.8%)	1395 (21.4%)	203 (22.9%)	1901 (25.9%)	107 (22.7%)	6245 (22.9%)
2013	3115 (25.8%)	1523 (23.4%)	210 (23.7%)	2548 (34.7%)	110 (23.4%)	7506 (27.5%)
**Male sex, n (%)**	6014 (49.8%)	3959 (60.7%)	463 (52.1%)	4709 (64.1%)	244 (51.8%)	15,389 (56.3%)
**Age–years**						
median (IQR)	36.9 (30.8–43.4)	37.1 (31.5–43.3)	37.4 (31.8–44.3)	36.3 (30.1–43.2)	36.4 (30.9–43.1)	36.8 (30.8–43.3)
**n (%)**						
18–34	4780 (39.6%)	2448 (37.6%)	322 (36.3%)	3048 (41.5%)	191 (40.6%)	10,789 (39.5%)
35–44	4676 (38.7%)	2668 (40.9%)	349 (39.3%)	2707 (36.8%)	185 (39.3%)	10,585 (38.8%)
45–59	2378 (19.7%)	1274 (19.5%)	189 (21.3%)	1429 (19.4%)	80 (17.0%)	5350 (19.6%)
≥60	250 (2.1%)	129 (2.0%)	28 (3.2%)	167 (2.3%)	15 (3.2%)	589 (2.2%)
**History of** **comorbidities, n (%)**						
Type 2 diabetes	230 (1.9%)	120 (1.8%)	12 (1.4%)	147 (2.0%)	12 (2.6%)	521 (1.9%)
Hypertension	459 (3.8%)	255 (3.9%)	39 (4.4%)	307 (4.2%)	14 (3.0%)	1074 (3.9%)
Ischemic heart disease	53 (0.4%)	33 (0.5%)	2 (0.2%)	61 (0.8%)	2 (0.4%)	151 (0.6%)
Tubulo-interstitial nephritis	258 (2.1%)	120 (1.8%)	16 (1.8%)	127 (1.7%)	6 (1.3%)	527 (1.9%)
Gout	42 (0.4%)	21 (0.3%)	5 (0.6%)	31 (0.4%)	1 (0.2%)	100 (0.4%)
Urolithiasis	78 (0.7%)	35 (0.5%)	3 (0.3%)	51 (0.7%)	3 (0.6%)	170 (0.6%)
Hepatitis B infection	92 (0.8%)	84 (1.3%)	69 (7.8%)	756 (10.3%)	33 (7.0%)	1034 (3.8%)
Hepatitis C infection	71 (0.6%)	128 (2.0%)	13 (1.5%)	252 (3.4%)	15 (3.2%)	479 (1.8%)
**Baseline absolute CD4 cell count**-cells/mm^3^ (n = 17,662)						
Median (IQR)	146 (49–244)	123 (39–250)	95 (33–248)	115 (37–253)	220 (65–399)	146 (49–244)
n (%)	8524	4194	454	4300	190	17,662
<200	5470 (64.2%)	308 (67.8%)	308 (67.8%)	2798 (65.1%)	89 (46.8%)	11,403 (64.6%)
≥200	3054 (35.8%)	1456 (34.7%)	146 (32.2%)	1502 (34.9%)	101 (53.2%)	6259 (35.4%)
**Variables with ≥20% missing values**						
Body mass index kg/m^2^ (n = 9266)						
Median (IQR)	20.8 (18.8–22.8)	20.0 (18.2–22.2)	20.6 (18.8–23.4)	20.2 (18.2–22.4)	20.4 (18.8–22.8)	20.4 (18.6–22.6)
n (%)	4528 (48.9%)	1916 (20.7%)	371 (4.0%)	2320 (25.0%)	131 (1.4%)	9266
<18.5	968 (21.1%)	1014 (52.9%)	174 (46.9%)	1180 (50.9%)	74 (56.5%)	2295 (24.8%)
18.5–22.9	2451 (54.1%)	549 (28.7%)	89 (24.0%)	662 (28.5%)	27 (20.6%)	4893 (52.8%)
23.0–24.9	557 (12.7%)	187 (9.8%)	53 (14.3%)	251 (10.8%)	18 (13.7%)	1086 (11.7%)
≥25.0	532 (11.8%)	166 (8.7%)	55 (14.8%)	227 (9.8%)	12 (9.2%)	992 (10.7%)
Baseline serum creatinine-mg/dL (n = 8524)						
Median (IQR)	0.90 (0.70–1.00)	0.85 (0.70–1.00)	0.83 (0.75–1.00)	0.86 (0.70–1.00)	0.79 (0.70–0.96)	0.82 (0.70–1.00)
Hyperlipidemia (n = 4868), n (%)	503 (21.6%)	231 (20.4%)	23 (17.3%)	203 (16.7%)	20 (35.1%)	980 (20.1%)

Abbreviations: 3TC, lamivudine; AZT, zidovudine; EFV, efavirenz; FTC, emtricitabine; LPV/r, lopinavir/ritonavir; NVP, nevirapine; TDF, tenofovir.

**Table 2:** Incidence per 1000 person-years of follow-up (PYFU) of chronic kidney disease according to the baseline characteristics among 27,313 HIV-infected adults who received one of the first-line antiretroviral therapy regimens followed for a median 2.3 years in the study.

	Number of Patients with CKD	PYFU	Incidence Per 1000 PYFU (95% CI)
**Overall**	245	76,168	3.2 (2.8–3.6)
**Fiscal year of** **antiretroviral initiation**			
2007	13	8846	1.4 (0.9–2.5)
2008	13	8366	1.6 (0.9–2.7)
2009	29	8660	3.3 (2.3–4.8)
2010	29	9908	2.9 (2.0–4.2)
2011	72	16,682	4.3 (3.4–5.4)
2012	51	13,435	3.8 (2.9–5.0)
2013	38	10,272	3.7 (2.7–5.1)
**Characteristics**			
**Sex**			
Female	111	35,757	3.1 (2.6–3.7)
Male	134	40,410	3.3 (2.8–3.9)
**Age**–years			
18–34	17	30,800	0.6 (0.3–0.9)
35–44	55	29,961	1.8 (1.4–2.4)
45–59	117	13,990	8.4 (7.0–10.0)
≥60	56	1416	39.5 (30.4–51.4)
**History of comorbidities**			
Type 2 diabetes			
No	217	75,068	2.9 (2.5–3.3)
Yes	28	1100	25.5 (17.6–36.9)
Hypertension			
No	205	73,942	2.8 (2.4–3.2)
Yes	40	2226	18.0 (13.2–24.5)
Ischemic heart disease			
No	242	75,867	3.2 (2.8–3.6)
Yes	3	301	10.0 (3.2–30.9)
Tubulo-interstitial nephritis			
No	234	74,961	3.1 (2.7–3.5)
Yes	11	1208	9.1 (5.0–16.4)
Gout			
No	240	75,983	3.2 (2.8–3.6)
Yes	5	186	26.9 (11.2–64.7)
Urolithiasis			
No	237	75,823	3.1 (2.8–3.6)
Yes	8	346	23.1 (11.6–46.3)
Hepatitis B infection			
No	234	73,931	3.2 (2.8–3.6)
Yes	11	2237	4.9 (2.7–8.9)
Hepatitis C infection			
No	236	75,115	3.1 (2.8–3.6)
Yes	9	1054	8.5 (4.4–16.4)
**Baseline absolute CD4 cell count**-cells/mm^3^			
<200	99	27,084	3.7 (3.0–4.5)
≥200	58	15,771	3.7 (2.8–4.8)
**First-line antiretroviral regimens**			
AZT + 3TC + NVP	79	34,130	2.3 (1.9–2.9)
AZT + 3TC + EFV	46	19,105	2.4 (1.8–3.2)
TDF + 3TC/FTC + EFV	80	18,889	4.2 (3.4–5.3)
TDF + 3TC + NVP	23	2599	8.8 (5.9–13.3)
TDF + 3TC + LPV/r	17	1445	11.8 (7.3–18.9)

Abbreviations: 3TC, lamivudine; AZT, zidovudine; CI, confidence interval; CKD, chronic kidney disease; EFV, efavirenz; FTC, emtricitabine; LPV/r, lopinavir/ritonavir; NVP, nevirapine; TDF, tenofovir.

**Table 3:** Factors associated with the risk of chronic kidney disease in HIV-infected adults who received currently recommended first-line antiretroviral therapy regimens (number of patients 27,313, except if otherwise specified).

Variables	Univariable ^a^		Multivariable ^a^	
	SHR (95% CI)	*p*-Value	aSHR (95% CI)	*p*-Value
**Fiscal year of antiretroviral initiation**				
2007	reference			
2008	1.0 (0.5–2.2)	0.993		
2009	1.7 (0.9–3.3)	0.133		
2010	0.9 (0.4–2.0)	0.813		
2011	1.5 (0.8–2.8)	0.217		
2012	1.1 (0.6–2.1)	0.827		
2013	0.8 (0.4–1.7)	0.565		
**Male sex**	0.9 (0.6–1.4)	0.662	0.9 (0.6–1.5)	0.779
**Age**–years				
18–34	reference		reference	
35–44	3.1 (1.8–5.4)	<0.001	2.9 (1.7–5.1)	<0.001
45–59	13.5 (8.1–22.5)	<0.001	11.6 (6.9–19.6)	<0.001
≥60	64.6 (37.4–111.6)	<0.001	47.6 (26.5–85.5)	<0.001
**Baseline history of comorbidities**				
Type 2 diabetes	6.6 (4.4–9.9)	<0.001	2.8 (1.7–4.5)	<0.001
Hypertension	4.9 (3.5–7.0)	<0.001	1.4 (0.9–2.1)	0.153
Ischemic heart disease	2.5 (0.8–7.7)	0.117		
Tubulo-interstitial nephritis	2.4 (1.3–4.5)	0.004		
Gout	6.4 (2.6–15.7)	<0.001	2.8 (1.1–7.0)	0.029
Urolithiasis	6.2 (3.0–12.8)	<0.001	3.6 (1.7–7.8)	0.001
Hepatitis B infection	0.6 (0.2–1.8)	0.391		
Hepatitis C infection	2.1 (0.8–5.5)	0.127		
**Baseline absolute CD4 cell count <200 cells/mm^3^**	0.9 (0.7–1.3)	0.572		
**First-line antiretroviral regimens**				
AZT + 3TC + NVP	reference		reference	
AZT + 3TC + EFV	1.1 (0.7–1.5)	0.712	1.0 (0.7–1.5)	0.973
TDF + 3TC/FTC + EFV	1.7 (1.2–2.3)	0.002	1.6 (1.2–2.3)	0.003
TDF + 3TC + NVP	4.1 (2.6–6.6)	<0.001	3.8 (2.3–6.0)	<0.001
TDF + 3TC + LPV/r	6.0 (3.5–10.2)	<0.001	6.5 (3.9–11.1)	<0.001
**Time-updated absolute CD4 cell count <200 cells/mm^3^** (n = 27,056)	2.1 (1.6–2.8)	<0.001	2.2 (1.7–2.9)	<0.001
**Variables with ≥20% missing values**				
**Baseline body mass index**-kg/m^2^ (n = 9266)				
<18.5	1.5 (0.9–2.5)	0.116		
18.5–22.9	reference			
23.0–24.9	0.9 (0.4–2.0)	0.883		
≥25.0	1.0 (0.5–2.2)	0.960		
**Time-updated HIV-1 RNA load ≥1000 copies/mL** (n = 27,056)	1.3 (0.8–2.1)	0.303		

Abbreviations: 3TC, lamivudine; aSHR, adjusted sub-hazard ratio; AZT, zidovudine; CI, confidence interval; EFV, efavirenz; FTC, emtricitabine; LPV/r, lopinavir/ritonavir; NVP, nevirapine; SHR, sub-hazard ratio; TDF, tenofovir. ^a^: The analysis was adjusted by the availability of previous serum creatinine measurement and propensity score.

## Data Availability

The data will not be shared because of patient confidentiality.

## Supplementary Materials

The following supporting information can be downloaded at: https://www.mdpi.com/article/10.3390/healthcare10081490/s1. Figure S1: Distribution of the study population according to the probability of each patient being assigned to a specific first-line antiretroviral regimen given their characteristics (propensity score). These graphs show the distribution of propensity score for each drug combination. The propensity score was calculated using multinomial (polytomous) logistic regression based on fiscal year, sex, age group, and history of comorbidities (hepatitis B and hepatitis C infection, hypertension, type 2 diabetes mellitus, ischemic heart disease, tubulo-interstitial nephritis, gout, and urolithiasis) at antiretroviral therapy initiation. Abbreviations: AZT, zidovudine; 3TC, lamivudine; TDF, tenofovir; FTC, emtricitabine; NVP, nevirapine; EFV, efavirenz; LPV/r, ritonavir-boosted lopinavir; Table S1: Incidence and cumulative incidence rate of chronic kidney disease in HIV-infected patients according to the duration of follow-up.
