# Supplementary material for: Chronic Kidney Disease in a Large National Human Immunodeficiency Virus Treatment Program
Source: Healthcare (Basel). 2022 Aug 8;10(8):1490. doi: 10.3390/healthcare10081490 (PMC9408286; doi:10.3390/healthcare10081490)
Supplement: Supplementary file 1 [file healthcare-10-01490-s001.zip › healthcare-1814800-supplementary.pdf]

Table S1. Incidence and cumulative incidence rate of chronic kidney disease in HIV-infected patients according to duration of follow-up

Incidence rate					Cumulative incidence rate				
**Duration of follow-up (years)**	**Number with chronic kidney disease diagnosis**	**Person-years of follow-up (PYFU)**	**Incidence rate per 1,000 PYFU**	**95% Confidence interval**	**Duration of follow-up (years)**	**Cumulative number with chronic kidney disease diagnosis**	**Cumulative person-years of follow-up (PYFU)**	**Cumulative incidence rate per 1,000 PYFU**	**95% Confidence interval**
0 to 1	95	26,177	3.6	3.0-4.4	0 to 1	95	26,177	3.6	3.0-4.4
1 to 2	76	20,353	3.7	3.0-4.7	0 to 2	171	46,530	3.7	3.2-4.3
2 to 3	42	13,067	3.2	2.4-4.4	0 to 3	213	59,597	3.6	3.1-4.1
3 to 4	16	7,788	2.1	1.3-3.4	0 to 4	229	67,385	3.4	3.0-3.9
4 to 5	8	4,352	1.8	0.9-3.7	0 to 5	237	71,738	3.3	2.9-3.8
5 to 6	3	2,639	1.1	0.4-3.5	0 to 6	240	74.377	3.2	2.8-3.7
6 to 7	4	1,379	2.9	1.1-7.7	0 to 7	244	75,755	3.2	2.8-3.6
7 to 8	1	413	2.4	0.3-17.2	0 to 8	245	76,168	3.2	2.8-3.6

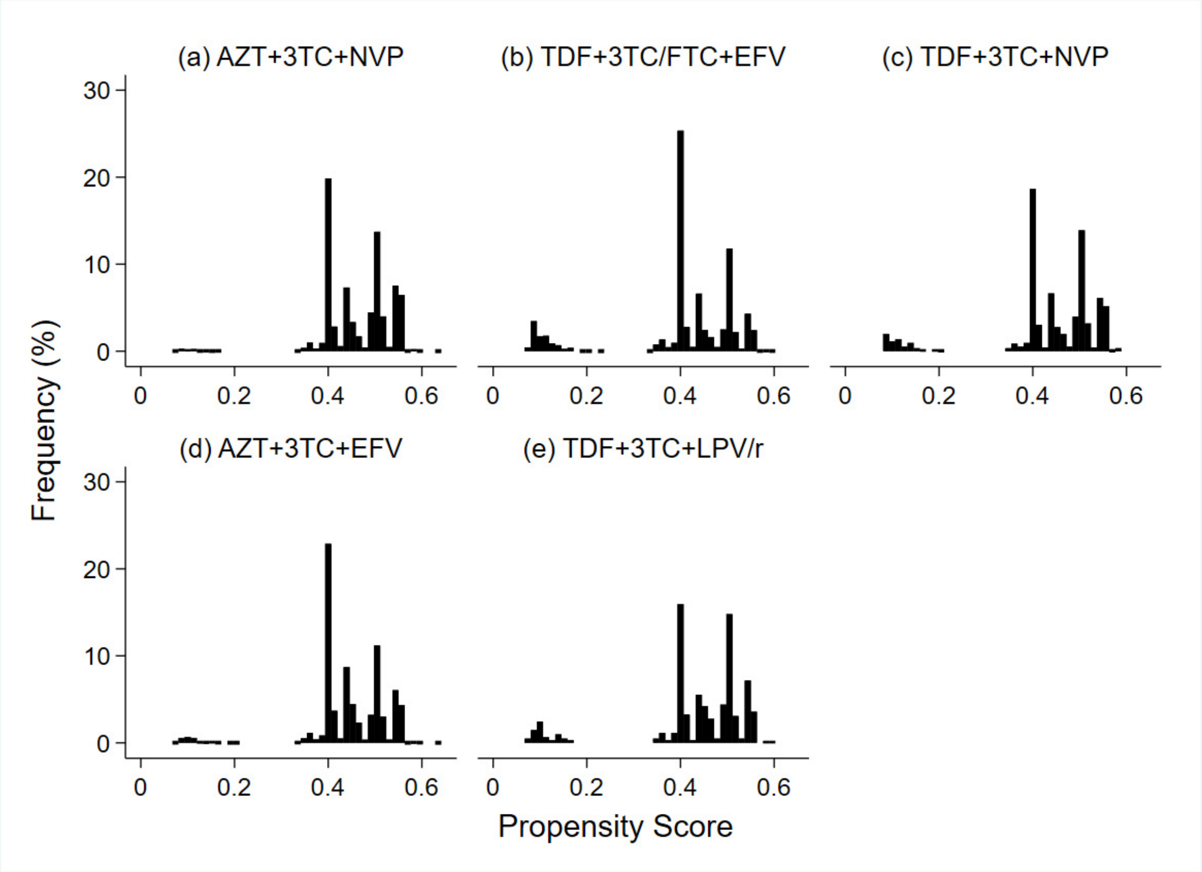

Figure S1. Distribution of the study population according to the probability of each patient being assigned to a specific first-line antiretroviral regimen given their characteristics (propensity score). These graphs show the distribution of propensity score for each drug combination. The propensity score was calculated using multinomial (polytomous) logistic regression based on fiscal year, sex, age group, and history of comorbidities (hepatitis B and hepatitis C infection, hypertension, type 2 diabetes mellitus, ischemic heart disease, tubulo-interstitial nephritis, gout, urolithiasis) at antiretroviral therapy initiation. Abbreviations: AZT, zidovudine; 3TC, lamivudine; TDF, tenofovir; FTC, emtricitabine; NVP, nevirapine; EFV, efavirenz; LPV/r, ritonavir-boosted lopinavir.
